# Early or synchronized gestures facilitate speech recall—a study based on motion capture data

**DOI:** 10.3389/fpsyg.2024.1345906

**Published:** 2024-03-25

**Authors:** Jens Nirme, Agneta Gulz, Magnus Haake, Marianne Gullberg

**Affiliations:** ^1^Lund University Cognitive Science, Lund, Sweden; ^2^Centre for Languages and Literature and Lund University Humanities Lab, Lund University, Lund, Sweden

**Keywords:** gesture, speech processing, multimodal integration, virtual animation, timing

## Abstract

**Introduction:**

Temporal co-ordination between speech and gestures has been thoroughly studied in natural production. In most cases gesture strokes precede or coincide with the stressed syllable in words that they are semantically associated with.

**Methods:**

To understand whether processing of speech and gestures is attuned to such temporal coordination, we investigated the effect of delaying, preposing or eliminating individual gestures on the memory for words in an experimental study in which 83 participants watched video sequences of naturalistic 3D-animated speakers generated based on motion capture data. A target word in the sequence appeared (a) with a gesture presented in its original position synchronized with speech, (b) temporally shifted 500 ms before or (c) after the original position, or (d) with the gesture eliminated. Participants were asked to retell the videos in a free recall task. The strength of recall was operationalized as the inclusion of the target word in the free recall.

**Results:**

Both eliminated and delayed gesture strokes resulted in reduced recall rates compared to synchronized strokes, whereas there was no difference between advanced (preposed) and synchronized strokes. An item-level analysis also showed that the greater the interval between the onsets of delayed strokes and stressed syllables in target words, the greater the negative effect was on recall.

**Discussion:**

These results indicate that speech-gesture synchrony affects memory for speech, and that temporal patterns that are common in production lead to the best recall. Importantly, the study also showcases a procedure for using motion capture-based 3D-animated speakers to create an experimental paradigm for the study of speech-gesture comprehension.

## 1 Introduction

Spontaneous manual gestures accompanying speech are prevalent in spoken communication. There is broad consensus that in production and reception of gestures and speech, the two modalities are coordinated both semantically and temporally (Kita and Özyürek, 2003; Kendon, 2004; de Ruiter, 2007; McNeill, 2008). More specifically, in speech production the expressive “stroke” phase (Kendon, 1980) of gestural movements tends to be aligned with elements in speech that are semantically or pragmatically associated with the gestural function (“lexical affiliates,” Schegloff, 1984; *cf*. “conceptual affiliates,” de Ruiter, 1998). This observed temporal alignment led McNeill (1992) to formulate the “phonological synchrony rule” stating that: “[a] gesture precedes or ends at, but does not follow, the phonological peak syllable of speech” (McNeill, 1992, p. 26). The rule has been empirically supported in both observational and experimental studies of speech and gesture production (*cf*. next section). However, the evidence regarding timing constraints in reception is sparser. It remains less clear whether and to what extent integration of speech and gesture information depends on the same pattern of synchronized or advanced^1^ gestures relative to speech. We addressed this question using a novel method where the timing of individual gesture strokes within naturally occurring speech-gesture sequences were manipulated and presented in 3D-animated digital speakers based on motion capture data from real speakers. We tested to what extent recall of associated words was affected by shifts in the temporal alignment of speech and gestures.

## 2 Background

### 2.1 Speech-gesture timing in production

The temporal relationship between speech and gestures in production has been the target of examination since the earliest days of gesture studies. Kendon (1980) showed that the expressive “stroke” phase of gestural movements tends to be aligned with or precede elements in speech that are semantically or pragmatically associated with its function. This observed temporal alignment led McNeill (1992) to formulate the “phonological synchrony rule” stating that: “[a] gesture precedes or ends at, but does not follow, the phonological peak syllable of speech” (McNeill, 1992, p. 26). The scope of the associated unit of speech may vary. Schegloff (1984) talked about “lexical affiliates” of gestures, emphasizing words, but de Ruiter (1998) proposed the term “conceptual affiliate” to capture the notion that gestures usually represent a whole idea, and do not necessarily relate to an individual word (*cf*. McNeill, 1992, 2008, p. 6–11).

Empirical work has explored the temporal relationships between speech and gesture in *production*, and the factors that influence this relationship. Seyfeddinipur and Kita (2001) showed that gesturing tends to stop or restart on average 240 ms before speech stops or restarts in disfluent sequences. Graziano and Gullberg (2018) similarly found that gesture strokes were mostly performed during fluent speech (and rarely during pauses or disfluencies). Other production studies have observed that gesture strokes tend to start before or simultaneously with semantically associated words and most often overlap, and are only rarely performed after the associated words, lending support to the phonological synchrony rule (Butterworth and Beattie, 1978; Ferré, 2010; Bergmann et al., 2011; Macuch Silva et al., 2020).

Levelt et al. (1985) and de Ruiter (1998) found evidence for coordination of speech and gestures taking place at the motor planning stage such that if one modality is disturbed, the other one adjusts. Similarly, Chu and Hagoort (2014) showed that the timing of speech and gesture is dynamically adjusted to maintain synchrony, e.g., speech onset is delayed if visual feedback of a gestural movement is artificially delayed. Relatedly, several studies have found that the timing of pointing strokes varies with the location of stress when manipulated (Esteve-Gibert and Prieto, 2013; Rusiewicz et al., 2013). Finally, synchrony has also been shown to be related to semantic redundancy between speech and gesture with redundant gestures being more tightly synchronized to speech than others (Bergmann et al., 2011), to word familiarity (Morrel-Samuels and Krauss, 1992), and that some parts of speech are more likely to synchronize with gestures (nouns and verbs) than others (Kok, 2017).

In sum, a wealth of studies of gesture production has shown that gestural strokes tend to occur before or with relevant units of speech, whatever those units are. This temporal pattern has also motivated the theoretical accounts of gesture production. Gestures occurring *after* relevant units of speech would not be helpful in these instances.

### 2.2 Speech-gesture timing in reception

There is now ample evidence in the literature that addressees attend to and process gestural information in reception (Kendon, 1994; Hostetter, 2011; Kelly, 2017). Kendon (1994) reviewed both experimental and observational studies investigating different ways in which gestures contribute to understanding, and concluded that although gestures are often non-essential to communication, there are many circumstances in which they do support both semantic and pragmatic understanding.

There are several mechanisms by which gestures might contribute to comprehension. One way in which representational gestures, which represent referent properties or actions, can affect reception is to complement speech with non-redundant information (size, direction, etc.). Several studies have found effects of non-redundant information in gestures on recall of semantic content (e.g., Beattie and Shovelton, 1999; Cassell et al., 2000; Church et al., 2007; Gullberg and Kita, 2009). Other studies have found that gestures can provide contextual cues (e.g., priming one interpretation of a word) in explicit discrimination tasks (Wu and Coulson, 2007; Yap et al., 2011) or implicit speech disambiguation (Holle and Gunter, 2007). Also, representational gestures that redundantly express similar information to the associated speech (e.g., pointing left and saying “left”) may improve recall by activating stronger or richer mental representations (e.g., Rogers, 1978; Riseborough, 1981; Woodall and Folger, 1985; Cohen and Otterbein, 1992; Kang and Tversky, 2016; Iani and Bucciarelli, 2017). Cook and Fenn (2017) reviewed effects of gestures on memory raising the possibility that seeing gestures while hearing speech may aid memory by reducing the cognitive load involved in processing, thereby freeing up working memory resources, as well as by strengthening and broadening encoding over several memory modalities. Finally, non-representational gestures such as beats can emphasize and draw attention to specific elements in speech (Krahmer and Swerts, 2007) and thereby affect processing (Biau and Soto-Faraco, 2021) and recall of speech (So et al., 2012; Kushch and Prieto Vives, 2016; Igualada et al., 2017).

In summary, gestures may inform listeners by expressing non-redundant information, support comprehension of speech by expressing redundant information, or highlight different aspects of speech. The weight of each function will likely vary between different listening situations. That being said, Hostetter (2011) summarized the results of 63 published studies on effects of representational gestures on listeners’ comprehension, memory, or learning, and found a medium overall effect of gestures no matter what type of an outcome measure was used. The meta-analysis also found that effects of non-redundant gestures were greater compared to redundant gestures.

### 2.3 The specific effects of speech-gesture timing on reception

Despite the many studies on speech-gesture coordination in production and reception reviewed above, relatively few studies have directly investigated effects of temporal coordination on reception (recall, ease of processing, and learning). The results are so far inconclusive.

Woodall and Burgoon (1981) found that listeners showed improved recall (but not recognition), persuasion and perceived credibility of the speaker after hearing a verbal argumentation accompanied by gestures performed by an actor in synchrony with “emphasized word clusters” compared to gestures lagging by around 1 s. A more recent study tested children’s learning from mathematical explanations by an “animated teaching agent” with gestures that had been scripted to align with speech or to be delayed or advanced by 500 ms. The authors found that delayed gestures were detrimental to learning (Pruner et al., 2016), leading to less improvement between pre- and post-tests of their understanding of the explained mathematical concepts. Anikin et al. (2015) compared participants’ ability to reconstruct configurations of geometric objects following verbal descriptions presented either as speech alone, as speech with gestures in three conditions: (1) in original synchrony or uniformly; (2) advanced; or (3) delayed by 1,500 ms. While the audio-only group performed significantly worse at reconstructing the configurations compared to the synchronized video group, the difference between the delayed and synchronized video groups was non-significant, and the advanced and synchronized performed equally well. The results indicate that participants were still able to benefit from spatial information in gestures (partly non-redundant relative to speech) even without synchrony. However, the uniform temporal manipulations offer limited control over how words and gestures ended up aligning and it is possible that participants adapted to the constant shift.

Neurocognitive techniques have also been used to study effects of the synchrony on speech-gesture integration. Biau et al. (2016) contrasted fMRI scans of participants watching video clips including synchronized beat gestures with conditions where the video track had been uniformly advanced by 800 ms and/or replaced with animated abstract corresponding to the hand movements of the beat gestures. They found differences in the BOLD signal localized to the left side middle temporal gyrus (MTG) and inferior frontal gyrus (IFG). EEG studies have found indications both of a fast and automatic mechanism and a slower and more demanding one. Using event-related potentials Habets et al. (2011) found that tight synchrony (between 160 and 360 ms) is a condition for automatic integration (indicated by modulation of the N400 signal). Kelly et al. (2010) found that congruent speech and gesture pairs (compared to incongruent) evoked reduced N400 effects and faster reaction times. This suggests a facilitating effect of gestures on semantic processing even when the explicit task is not related to comprehension (deciding gender of a speaker). Other evidence is indicative of slower, intentional integration. Obermeier and Gunter (2015) found N400 indications of automatic integration only when gestures started within ±200 ms relative to the word. However, the N400s related to a later disambiguating word were reduced after seeing congruent compared to incongruent gestures also when gestures preceded words by as much as 600 ms, indicating that gesture information was still accessible despite the asynchrony with the earlier word. Kelly et al. (2007) found different loci of N400 effects comparing congruent and incongruent gestures depending on whether participants had been told gestures and speech were produced by same person, indicating a top-down influence on integration.

Other lines of research have investigated effects of temporal coordination of gestures that are only indirectly related to speech reception. Words that were temporally overlapping with beat gestures have been found to be perceived as more strongly emphasized (Krahmer and Swerts, 2007; Treffner et al., 2008). Bosker and Peeters (2021) showed that such a shift of the perceived stress (what they call the “manual McGurk effect”) can affect the disambiguation of words (e.g., obJECT and OBject). Others have focused on the perception of the gestures themselves. Kirchhof (2014) tested explicit detection of asynchronous audio and video tracks of videos showing co-speech gestures, and found tolerance for temporal offsets by as much as 600 ms in either direction in 60% of the trials. Dimitrova et al. (2016) found that beat gesture strokes that started 520 ms before an associated word, making the apex of the stroke aligning with the onset of the word, were subjectively judged as the most natural. Similarly, Esteve Gibert et al. (2014) found that pointing gestures where the apex coincided with an unstressed syllable were judged to be less natural compared to when coinciding with a stressed syllable. In contrast, Nirme et al. (2019) found no significant effect on perceived naturalness of individual representational gesture strokes that had been either advanced or delayed by 500 ms in a judgment task with a less explicit focus on gestures.

In contrast to these studies, others highlight that gestures inform speech processing beyond individual words. Cutica and Bucciarelli (2008) found improved verbatim and paraphrased recall of events within verbal narratives, but also a stronger tendency to misrecognize paraphrases as verbatim when accompanied by (redundant) gestures. The authors interpreted their results as indicating that gestures inform a “deeper” mental model, less connected to the “superficial” verbal content. Relatedly, Kelly (2017) found that while non-representational gestures may highlight words within a sentence, they do not contribute to perceived emphasis within a word.

In summary, results from studies investigating the specific effects of speech-gesture (a) synchrony are inconclusive. In some cases, small offsets seem to matter, in others they do not. Despite the body of work reviewed above, it still remains unclear how sensitive the speech-gesture integration process is to asynchrony in general, and specifically to violations of the phonological synchrony rule stating that natural gesture strokes are produced before or during, but not following speech. Little is known about whether effects of advancing or delaying gestures has a symmetrical effect on recall. Further clarifying this can provide important insights into how speech and gestures are integrated and further, whether speech-gesture integration is conditioned by the timing characteristics of natural production. Importantly, a possible reason for the inconclusiveness of these studies is that methods vary across studies, and that it is challenging to design studies that manipulate the speech-gesture alignment without also tampering with other factors in the input. For this reason, the current study addresses these issues by suggesting a paradigm built on motion capture recorded natural gestures embedded in stretches of natural discourse, which have then been turned into 3D-animated speakers (*cf*. Nirme et al., 2019).

### 2.4 Research questions and predictions

The current study investigates effects on speech recall of temporally shifting (delaying or advancing) or eliminating accompanying gestures. We ask the following questions:

(1) Do synchronized gestures strengthen memory for co-occurring words? We predict that words accompanied by synchronized gesture strokes are more likely to be remembered, indicative of stronger multimodal meaning encoding.
(2) Do gestures that start ahead of their associated words have the same effect on recall as synchronized gestures (since early starts is common in spontaneous production)? We predict that words whose related gesture strokes occur earlier than the related speech, will be as likely to be recalled as synchronized gestures.
(3) Do gestures that are delayed relative to their associated words (rare in spontaneous production) weaken memory encoding compared to synchronized gesture strokes? We predict that words whose related gesture strokes are delayed relative to speech are less likely to be remembered than words accompanied by synchronized gesture strokes.

## 3 Materials and methods

### 3.1 Design

To address the research questions, we conducted an experiment in a between-subject design where we tested participants’ tendency to include target words in verbal reproductions of speech that had appeared in short 3D-animated video clips. Target gestures, recorded with motion capture from real speakers and then reproduced as animated 3D characters, were shown associated with target words, embedded in stretches of authentic speech production. The target gestures were either presented (a) in original synchrony with spontaneous speech (G-SYNC), (b) advanced (G-ADV), or (c) delayed by 500 ms (G-DELAY), or (d) eliminated entirely (NO-G). Participants were randomly assigned to one of these conditions and were tasked with verbally reproducing what they had heard in the videos as accurately (verbatim) as possible. Their responses were scored for inclusion of the target words.

Traditionally, psycholinguistics studies suggest that language users are poor at verbatim recall, especially of syntactic structure in comprehension (e.g., Sachs, 1967; Gernsbacher, 1985; Loebell and Bock, 2003, *inter alia*). However, more recent findings suggest that memory for syntactic structure may be relatively good under certain circumstances (e.g., Gurevich et al., 2010). More importantly, since we are testing the recollection of meaning and words—not syntax—we believe that this is a viable option for testing recall.

### 3.2 Participants

Eighty-three participants (44 female; *M*^age^ = 23, *SD* = 3) were recruited among students or faculty members around Lund University campus and randomly assigned to one of four groups (defined by the four conditions). Each participant received a voucher of 40 SEK to spend in the campus cafeteria. Two participants were excluded; one due to failure to comply with the instructions, and the other due to reporting that speech was not distinguishable in the video stimuli.

The number of participants, gender and age per group was distributed as follows: G-SYNC: *N* = 19 (11 female), age M = 23.9 (*SD* = 3.7); G-ADV: *N* = 20 (14 female), age *M* = 23.7 (*SD* = 2.9); G-DELAY: *N* = 19 (10 female), age *M* = 23.1 (*SD* = 1.5); NO-G: *N* = 23 (9 female), age *M* = 23.6 (*SD* = 4.1). A Kruskal–Wallis test found no age differences between the groups (*H* = 1.26, *p* = 0.74), and a Chi-square test found no differences in gender distribution (χ2 = 4.24, *p* = 0.24). The majority of participants were undergraduate students, and sampled from the same pool with random group assignment. The participants’ educational background was not further probed (i.e., area of study, year of study, etc.).

### 3.3 Materials

#### 3.3.1 Stimuli

**Figure 1:**
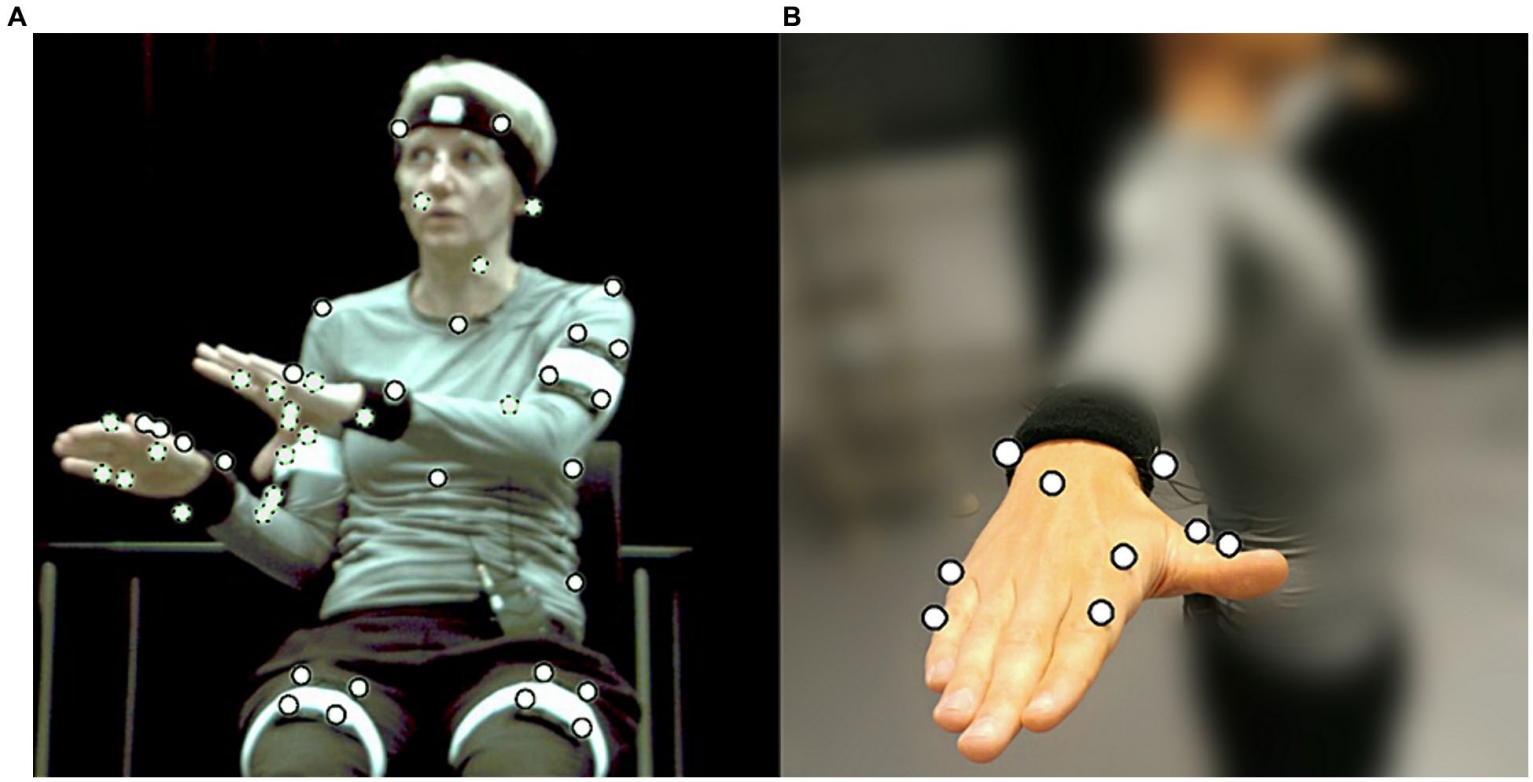
**(A)** Speaker during MOCAP recording wearing markers (white circles). Dotted outlines indicate markers concealed in the current view. **(B)** Placement of MOCAP markers on hands and fingers.

To create speech-gesture stimuli in which we could manipulate gestural temporal alignment keeping all other things equal, we worked with digitally animated renditions of speakers based on motion capture (MOCAP) of real speakers producing spontaneous speech and gestures. Two speakers (one male, one female) were recorded with a *Qualisys* system as they were spontaneously describing objects, cartoon or film excerpts, or route descriptions (in total 10 descriptions) to a confederate conversational partner. The speakers were volunteers who were unaware of the interest in their gestures during the recordings. They were debriefed after the recordings. Speakers were seated on a chair without armrests and spoke to a confederate sitting approximately 2 m in front of them. In total 25 MOCAP markers were placed on the torso, head, legs, arms and feet of the speakers (Figure 1A). Seven additional markers were placed on each hand to capture hand configuration (Figure 1B). The three-dimensional movement of the markers was recorded by 8 infrared cameras (Qualisys ProReflex MCU240) at 100 frames per second. Speakers’ faces were recorded by an Xtion Pro integrated depth sensor and camera at 30 frames per second. Lip movement, facial expressions, rudimentary gaze direction and eye blinks were subsequently extracted in FaceShift Studio (2015). Audio was recorded using a ZOOM H4 Handy Recorder. Audio and video were synchronized by a clapping board fitted with markers at the start of each recording.

In post processing, marker trajectories (3D positions over time) were calculated, labeled, and reconstructed where necessary using Qualisys Track Manager (version 2.10, 2015). The labeled marker trajectories were imported into Autodesk Motion builder (version 2014, 2013) and used to calculate (“solve”) joint rotation of a 3D-model matching the respective speaker’s gender and proportions. Autodesk Maya (version 2014, 2014) was used to combine joint rotations and facial animation and render 1,024 × 768 pixel images from approximate viewpoint of the addressee’s head position at a frame rate of 25 fps. Images were combined with audio into video files using the *Avidemux* video editor software (version 2.5.2, 2009).

From the recordings, 16 short excerpts (*M* duration = 9.51 s, *SD* = 1.80 s) were selected to be rendered as animations (7 with the female speaker). The selected excerpts fulfilled the following criteria: (1) the MOCAP and rendered animations were of sufficient quality and faithfully captured the gestures; (2) it included a *target gesture stroke* that temporally overlapped with the stressed syllable of a word that then became the *target word* (determined by frame-by-frame analysis using the ELAN software^2^; Wittenburg et al., 2006); (3) the target word was included in an utterance describing an event and the excerpt included at least one more gesture apart from the target; (4) there was a minimum of 1 s between the target gesture stroke and a preceding or following gesture. No gestures coincided with a disfluency. See Supplementary material for a complete list of the 16 items and brief descriptions of the gestures, transcribed by the first author as well as normalized frequencies of target words in corpora of Swedish news and magazine texts.

Selected target gestures had a mean stroke duration of 0.38 s (*SD* = 0.14 s). To ascertain whether it was possible to deduce target words by observing the target gestures alone, we asked two raters to guess what word was associated with the target gestures presented in isolation and with the speaker’s face blurred. Neither rater correctly identified any of the associated words.

**Figure 2:**
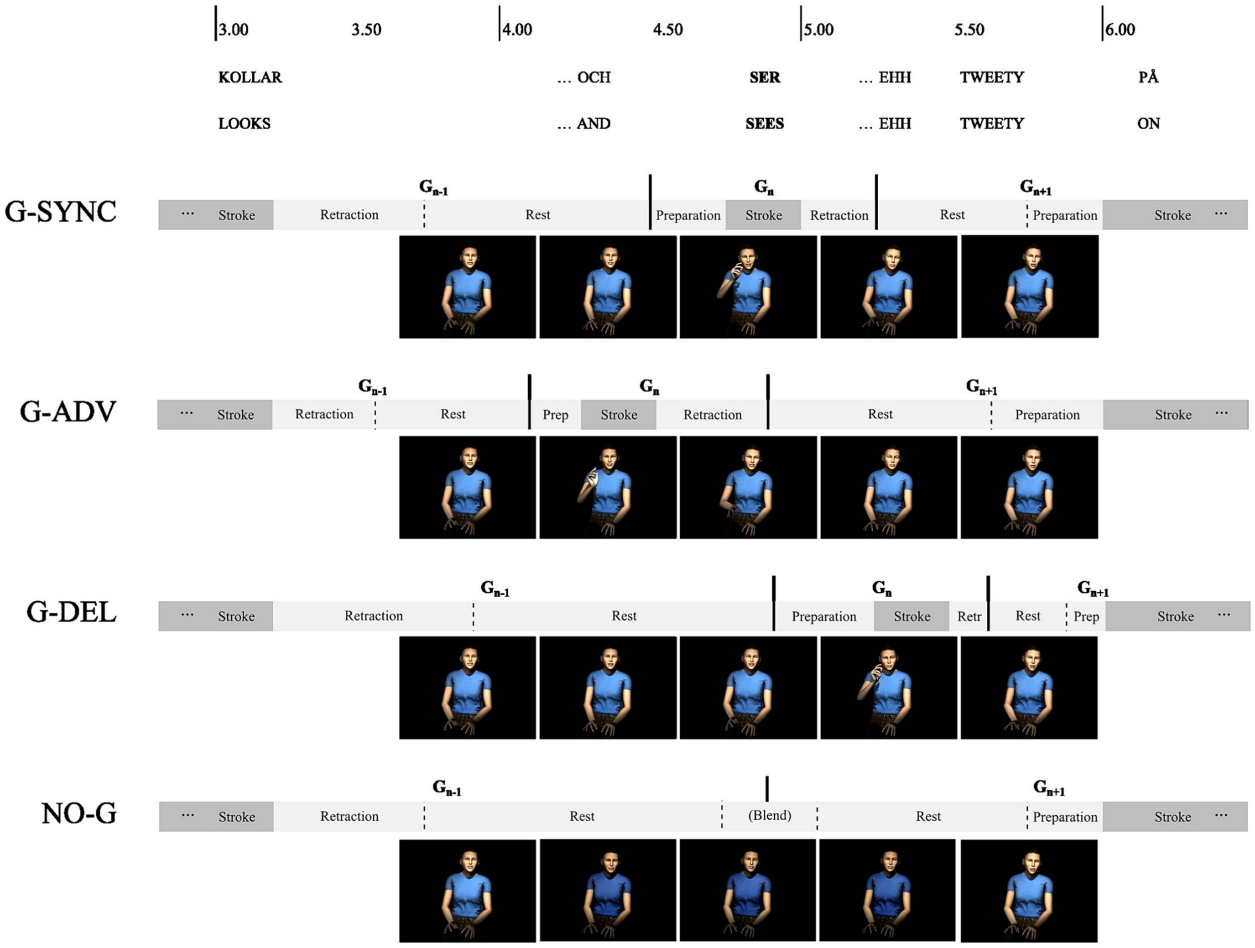
Timeline illustrating gesture manipulations, advancing, delaying, or eliminating target gesture (*G*_n_) strokes by speeding up or slowing down the preceding and following movement phases.

The resulting 16 selected video clips were used to create four conditions: two control and two experimental conditions. The original stimuli set with synchronized target gestures and target words were used as stimuli in the first control condition (G-SYNC). A second control condition was established by creating 16 new video clips from rendered images where the target gesture had been eliminated by speeding up, slowing down and blending together surrounding gesture phases (see Figure 2, NO-G condition). A first experimental condition was created by rendering 16 video clips where the 3D-animated stroke of the target gesture had been advanced by 500 ms relative to its original location by speeding up and slowing down surrounding preparation-, retraction-, hold-, or resting- phases (see Figure 2, the G-ADV condition). The duration of target gesture strokes was preserved whereas their onsets and offsets were advanced. Audio and facial animation were not affected by these manipulations and remained identical to the videos in the G-SYNC condition. Finally, a second experimental condition was based on 16 rendered video clips where the 3D-animated stroke of the target gesture had been delayed by 500 ms relative to its original timing (see Figure 2, the G-DELAY condition). Figure 2 illustrates the manipulations in one of the videos. Note that the manipulations were made on the 3D-animations, not the rendered videos, and that facial animations including lip sync preserved the original synchronization to audio throughout the clips in all conditions. Audio levels were Root Mean Square normalized in Audacity (version 2.1.1, 2015). For a more detailed description of the clips, manipulations and recording procedure, see Nirme et al. (2019). Examples of videos including synchronized, advanced, and delayed gestures can be found in on the Open Science Framework.^3^ Nineteen participants were pseudo-randomly assigned to the G-SYNC condition, 23 to the NO-G condition, 20 to the G-ADV condition, and 19 to the G-DELAY condition.

#### 3.3.2 Recall task

In the main experimental task participants watched video clips and were then asked to repeat verbally, as accurately as possible, what they had heard the speakers say in each clip.

#### 3.3.3 Buffer task

A simple buffer task was created, with the purpose of preventing participants to rely on the phonological loop in working memory (Baddeley, 1992) or explicit strategies to (subvocally) repeat what they had heard. The task consisted of reading out loud a list of 10 four-digit numbers displayed on the screen. This task was performed after each experimental trial.

#### 3.3.4 Experimental platform

The experimental platform, within which the stimuli in the experimental and the buffer task were presented and data was collected, was implemented as a browser-based application. The front-end was developed in HTML and JavaScript, and the backend used the Django framework (version 1.8, 2015). The application was executed in the Chrome web browser (version 43.0.2357, 2015) with the server running locally on a PC Windows computer. Verbal reproductions were automatically saved as an MP3 file marked for participant ID, trial, and condition in a MySQL database.

### 3.4 Procedure

Participants were tested individually in a small room. They provided written consent and were then seated on a chair approximately 2 m in front of a 160 × 120 cm projector screen. Participants were positioned as to approximate the typical face-to-face view of the speaker, such that their gaze line aligned with the horizontal center and 30 cm above the vertical center of the screen.

The screen initially displayed a welcome message and instructions that they would be presented with a series of short video clips of a digitally animated character speaking. The instructions did not include any mention of gestures or target words. They did say that participants were going to be asked to repeat everything they heard speakers say as accurately as possible after each clip. Participants were also asked to enter their gender and age into the respective fields on the computer, using a mouse and keyboard. When they pressed submit, their inputs were logged via the browser-based application running the experiment. The software also (pseudo-) randomly assigned each participant to one of the conditions; G-SYNC, G-ADV, G-DELAY, or NO-G. Mouse and keyboard were then removed, and participants were asked to wear a Sennheiser PC 363D headset and encouraged to adjust it to comfortably cover their ears.

The experimenter was seated on the side of the screen for the remainder of the procedure facing the participant in order not to see what was displayed. The experimenter ensured that participants were in fact watching the screen. A practice trial was started when participants indicated that they were ready by the experimenter pressing the space key on a keyboard. The practice trial started with a short video (of comparable duration but not included in the 16 experimental stimuli) including speech and gestures in original synchrony followed by the buffer task.

Having finished the buffer task, participants were instructed to verbally reproduce as accurately as possible what the speaker in the video had said into the microphone of the headset. At the end of the practice trial the experimenter asked the participant to confirm that they had been able to hear the speech in the video and that they understood the task instructions.

When participants were ready, the experimenter started the first experimental trial by pressing the space bar on the keyboard. The experimental trials followed the same format as the practice trial: video presentation followed by the buffer task and the verbal reproduction task, except that the version of the stimulus videos corresponded to the condition they had been assigned to. Between each trial participants confirmed that they were ready to proceed before the experimenter triggered the next trial. The order of the stimulus videos was randomized.

After completing all 16 trials, participants were told to remove the headphones and received new instructions to fill in a short questionnaire on-screen using a mouse. The questionnaire consisted of two ratings on visual analog scales presented as horizontal sliders on the screen. The first rating was in response to the question *“How much of what was said in the videos did you try to remember by repeating it to yourself*.*”* This was included to control that the buffer task had been effective, and there was no bias of a repetition strategy affecting the result. The second rating was in response to the question “How much of what was said in the videos did you find consistent with how the speaker moved.” This was included to check if participants became aware of the manipulations of the target gestures over the course of the experiment. Both scales ranged from 0% (at the leftmost end) to 100% (at the rightmost end), with intermediate ticks.

### 3.5 Data coding

All audio recordings of the participants’ verbal reproductions were coded by two independent coders blind to the conditions using a customized interface. For each case, coders saw the correct transcript of the speech (in the videos) to be reproduced in the trial, with the target word highlighted, a participant ID and link to the audio file, with no reference to the condition. One of the coders was familiar with the purpose of the study, while the other was not. Coders listened to the participants’ audio recordings, and compared them to the transcripts.

Accurate recall of the target word in each verbal reproduction was defined as (1) verbatim mention of the target word within the correct event (verbatim recall), or (2) verbatim or paraphrased mention of the meaning of the target word within the correct event (gist recall). For example, recollections of the utterance in (1) (target word in capitals) were coded as verbatim recall if they contained the actual target word (e.g., “*… then he DROPS his stuff*”), but as gist recall if they contained a related meaning (e.g., “*his things fall to the ground*”).

(1) *och så blir han typ omkullknuffad*, *TAPPAR sina grejer som han bär på* … *han fortsätter cykla*.

‘and then he gets kind of pushed over, DROPS his things that he is carrying… he carries on cycling.’

All responses coded as verbatim recall scores were thus also coded positively for gist recall, but not necessarily the inverse. Recollections that were neither coded as verbatim nor gist recall, were coded as non-recall. Overall accuracy of the reproduction was not scored since we were interested in specific effects on encoding of the target gestures, although the experimental task had no explicit focus on target words.

The scores of the two coders were compared and agreement deemed adequate for both gist recall (Cohen’s kappa = 0.91) and verbatim recall (Cohen’s kappa = 0.95). Entries with disagreement (69 data points, 5.3% of total) were excluded from further treatment and analysis.

Questionnaire responses were logged as values ranging between 0 and 1 with 3 decimal points, without transformation.

### 3.6 Data treatment and analysis

#### 3.6.1 Between group effects

The scores resulting from the coding were subjected to statistical analysis. We analyzed the verbatim recall and the gist recall scores separately. Both scores were aggregated per participant. The main hypothesis testing was made by linear regression, modeling recall accuracy score as a linear model with condition as fixed factor, with G-SYNC as reference level.

#### 3.6.2 Item level analysis (*post hoc*)

Although the temporal shifts defining conditions G-ADV and G-DELAY were always 500 ms in either direction, the target stroke duration and exact difference between target stroke onset and the onset of the stressed syllable in the target word varied between items. The resulting interval between stressed syllables in target words and advanced/delayed target gesture stroke thus varied between items. The mean interval for the items in the G-ADV condition was 0.63 s (*SD* = 0.13 s) and in the G-DELAY condition 0.37 s (*SD* = 0.13 s). The greater intervals in the G-ADV condition were due to gesture stroke onsets preceding the stressed syllables in target words (by definition of selection criteria). Also, six of the 32 temporally shifted target gestures, five in the G- ADV condition and two in the G-DELAY condition, ended up overlapping with pauses in speech. A previous study had found that the latter cases were perceived as less natural and as possibly drawing undue attention to the gestures (Nirme et al., 2019).

To clarify whether these factors influenced the result, we performed an item level analysis, this time excluding the NO-G condition. We ran a mixed-model logistic regression analysis, modeling the log-odds for positive recall scores (0 or 1) for each item as a linear combination of the fixed continuous factors INTERVAL-ADV (intervals between onsets of stressed syllable in target words and onsets of advanced target strokes), INTERVAL-DEL (intervals between onsets of stressed syllables in target words and onsets of delayed target strokes), a fixed categorical factor G-DURING-PAUSE (1 for target gesture strokes that overlap with a pauses, otherwise 0), and random factors item and participant.

Questionnaire responses were not normally distributed and analyzed by non-parametric tests (Kruskal–Wallis rank sum and Spearman’s rank correlation tests).

All statistical analyses were performed in R (version 3.2.5, R Core Team, 2016). We performed mixed-model logistic regression analyses using the lmerTest package (Kuznetsova et al., 2015) and coefficients of determination for mixed models (conditional *R*^2^, Nakagawa and Schielzeth, 2013) using the MuMIn package (Barton, 2013).

## 4 Results

### 4.1 Between group effects

**Figure 3:**
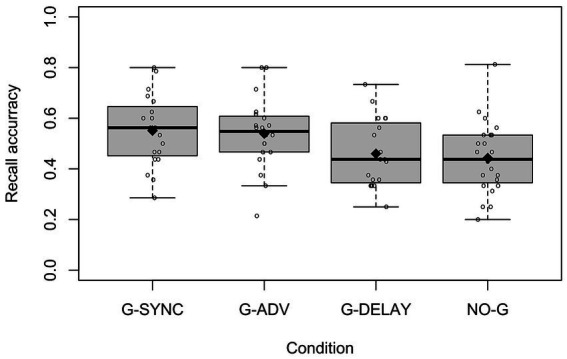
Distributions of verbatim recall accuracy per condition. ♦ = condition means, = recall accuracy per participant.

To test our predictions, we compared mean recall scores per participant in the G-SYNC condition to the other three conditions. Figure 3 presents the distribution of accurate recall scores. Although gist recall scores are by definition greater or equal to verbatim recall scores, there was a strong linear correlation (Pearson’s *r*: 0.92, *p* < 2.2e-16) between the two measures. The two scores were only different in 6.0% of cases. Therefore, we limited our further analysis to verbatim recall where we had stronger inter-coder reliability.

The mean verbatim recall accuracy (proportions) was 0.551 (*SD* = 0.143) in the G-SYNC condition, 0.443 (*SD* = 0.140) in the NO-G condition, 0.539 (*SD* = 0.142) in the G-ADV condition, and 0.460 (*SD* = 0.136) in the G-DELAY condition. The linear regression revealed that condition accounted for about 11% of the variance of verbatim recall, *R^2^* = 0.108, *F*(3,77) = 3.106, *p* = 0.031. Recall for G-SYNC was significantly higher than for NO-G (*β* = −0.108, *p* = 0.015) and G-DELAY (*β* = −0.091, *p* = 0.049), but there was no difference in recall between G-SYNC and G-ADV (*β* = −0.012, *p* = 0.798).

### 4.2 Item level analysis (*post hoc*)

**Figure 4:**
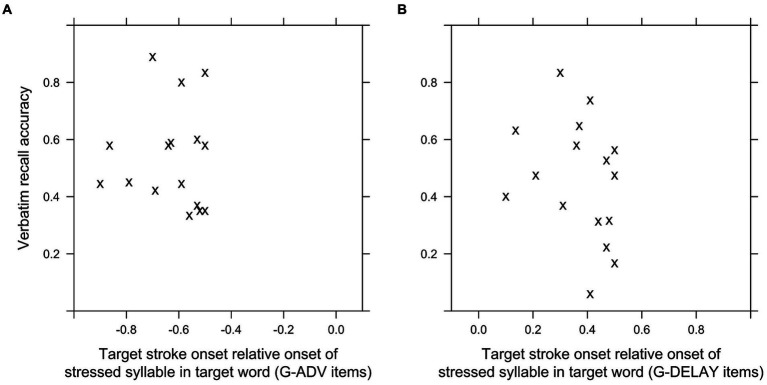
Verbatim recall accuracy per item, plotted against the interval between target strokes before **(A)** and after **(B)** target words.

To investigate how the durations of intervals between advanced or delayed target gestures and target words and eventual overlap with pauses in speech affected recall, we analyzed verbatim recall at the item level (see section 3.8). Fixed factors INTERVAL-ADV, INTERVAL-DEL and G-WITH-PAUSE with random factors item and participant accounted for about 17% of the variance of the log odds of verbatim recall (conditional *R^2^* = 0.173). INTRV-ADV had no significant relationship to verbatim recall accuracy (*β* = −0.312, *p* = 0.282), in line with the result of the between-group analysis. INTRV-AFTER had a significantly negative relationship to verbatim recall accuracy (*β* = −1.184, *p* = 0.013), also in line with the result of the between-group analysis. The relationship between G-W-PAUSE and verbatim recall accuracy was non-significant (*β* = 0.443, *p* = 0.063). Figure 3 shows the mean gist- and verbatim recall accuracy per item plotted against the intervals in G-ADV and G-DELAY conditions. There was an apparent tendency for greater intervals between target words and delayed target gestures to be associated with reduced recall of target words, with a sharp decline after 400 ms (Figure 4B). No such tendency was apparent for target gestures advanced to appear before the target word (Figure 4A).

### 4.3 Questionnaire results

To check whether outcomes were biased by participants sub-vocally repeating the speech content, we analyzed their responses to the question *“How much of what was said in the videos did you try to remember by repeating it to yourself”* (percentage entered on a visual analog scale). A Kruskal–Wallis rank sum test revealed no significant differences between the ratings in the different conditions (*H* = 5.15, *p* = 0.161). Further, a Spearman’s rank correlation test revealed no significant correlation between rating and verbatim recall (*r* = 0.160, *p* = 0.154).

To check whether participants had become aware of the manipulations of the target gestures, we analyzed their responses to the question “*How much of what was said in the videos did you find consistent with how the speaker moved”* (percentage entered on a visual analog scale). A Kruskal–Wallis rank sum test revealed no significant differences between the ratings in the different conditions (*H* = 0.652, *p* = 0.884).

## 5 Discussion

This study investigated whether gestures performed in synchrony with target words in speech enhance the recall of these spoken words, and whether recall is affected by gesture strokes occurring before the target word (common in gesture production) or after (rarer in gesture production). We measured the rate of verbatim recall of target words in verbal recollections following a buffer task.

The results revealed that, as predicted, synchronized target gestures made target words more likely to be remembered verbatim compared to when the same sequence was presented without the target gesture, or when the target gesture had been delayed by 500 ms. There was no difference in recall when the target gesture had been advanced by 500 ms.

### 5.1 Early or synchronized gestures aid recall

Our results are in line with previous research showing facilitating effects on comprehension or subsequent recall when gestures redundantly accompany speech (Kendon, 1994; Hostetter, 2011; Kelly, 2017). The improved verbatim recall of target words seemingly contradicts the finding of impaired recognition of verbatim sentences by Cutica and Bucciarelli (2008). However, it is possible that the tasks in the two studies, free recall and recognition, pose different demands on encoding (see discussion in Galati and Samuel, 2011).

So why should synchronized gestures help recall? As indicated above, one possible mechanism through which synchronized gestures may improve recall is by adding emphasis and drawing attention to associated words (So et al., 2012; Kushch and Prieto Vives, 2016; Igualada et al., 2017). However, previous work indicates that representational gestures have a more pronounced effect on both comprehension (Kang et al., 2013) and recall (Woodall and Folger, 1981, 1985; Feyereisen, 2006) compared to non-representational gestures. While Treffner et al. (2008) found that the positive effect on perceived emphasis diminished when gestures where both delayed or advanced, the current study only revealed significantly reduced recall by delayed but not advanced gestures, contradicting that the effect of synchronized gestures on recall would be driven by emphasis.

Another possible mechanism concerns gist redundancy by which gestures representing the same information as the target words redundantly elicit a multimodal representation that better serves retrieval. Our results are in line with such accounts (e.g., Church et al., 2007). However, the contribution of this mechanism may be related to how participants approached the recall task. Ackerman (1981) found that instructions to focus on items in a memory task on a unimodal sensory level or on a multimodal, semantic level made adult participants encode the information accordingly. We did not give any such explicit instructions, but since participants were told to watch the videos, and all videos had gestures, there may have been an implication to approach the task multimodally that may or may not be present in a real-world listening situation.

A final possibility is that gestures provide non-redundant contextual cues (e.g., Holle and Gunter, 2007). Some of the included target gestures could be said to express non-redundant information. One example was the gesture associated with the word *fastnar* “stuck” in *så fastnar han med hjulet* “he gets stuck with the wheel.” The speaker performed a gesture in character-viewpoint as if holding handlebars, thus revealing that it is the front wheel of a bike. However, in the context of the recall task, it is hard to define the exact contribution of this information. It is not directly crucial to recall the word or concept of getting stuck specifically. Therefore, the result of the current study does not allow us to isolate the contribution of non-redundancy.

To disentangle the possible explanations, a follow-up study should include a wider range of gesture stimuli, with distinct categories of representational (redundant and non-redundant) and non-representational gesture.

### 5.2 Late gestures do not aid recall

The results also show a similar advantage for recall when target gestures were advanced by 500 ms relative to their original timing, consistent with our prediction. In contrast, verbatim recall was less likely when target gestures had been delayed by 500 ms compared to synchronized target gestures. This is in line with our prediction, as well as previous findings that an “animated teaching agent” with delayed scripted gestures was detrimental to learning compared to one with synchronized gestures (Pruner et al., 2016).

A possible explanation for the reduced recall of words associated with delayed gestures, is that delayed gestures (as opposed to advanced or synchronized gesture) do not prepare listeners to semantically process words (Cook and Fenn, 2017). Yap et al. (2011) found faster response times on a lexical decision task when words were preceded by a video showing related compared to unrelated gestures without audio. Similarly, neurocognitive findings indicate that delayed speech make semantic integration less automatic (Habets et al., 2011; Obermeier and Gunter, 2015), and therefore presumably more effortful. The addition of a buffer task or distracting background noise to increase the demands during encoding in the current study could possibly also have resulted in reduced recall with advanced gestures. Several participants reported that the recall phase of the experimental task (to repeat all the speech in the stimulus videos) had been more challenging than expected. However, only one (excluded) participant reported difficulties hearing and understanding what the speakers said.

Since delayed gestures are atypical in natural speech-gesture production (Ferré, 2010; Bergmann et al., 2011), and no negative effect was found for advanced gestures, another possible explanation for the negative effect on recall of delayed gestures is that an unfamiliar multimodal stimulus pair is less likely to be integrated. The finding by Dargue and Sweller (2018) that atypical but congruent gestures do not facilitate comprehension as much as congruent typical gestures supports this explanation. On the other hand, an atypical speech-gesture pairing might increase participants’ attention to a particular segment of speech (Galati and Samuel, 2011, p. 430; Straube et al., 2014). The positive (but non-significant) effect (*p* = 0.063) of target gestures performed during pauses in speech makes us hesitant to rule out that the inclusion of those cases might have affected the outcome. Production studies have found that gestures tend not to co-occur with pauses (Graziano and Gullberg, 2018), and therefore such gestures may have drawn undue attention. A previous study using the same temporally shifted stimulus material found that participants were more likely to perceive clips with gesture strokes during pauses as unnatural (Nirme et al., 2019), but found no effect on perceived naturalness of temporal offset *per se*.

The results also relate to the broader question of whether the integration of speech and gestures is special, and follows the proposed fundamental link between speech and gesture in production (Kita and Özyürek, 2003; Kendon, 2004; de Ruiter, 2007; McNeill, 2008), or whether it is one example of general multimodal integration [see discussion in Kelly (2017), p 243]. Our results suggest that integration of co-speech gestures in comprehension is attuned to their coordination in production, with tolerance for gestures seen before but not after the target words. Although physical prerequisites dictate that light travels faster than sound, a 500 ms interval between detection of the same event by sight and hearing (which our results indicate still supported integration) cannot be assumed to support multimodal integration in general. Our findings thus lend support for the former position: that integration of speech and gestures is special in as much as it is attuned to the temporal coordination in production, not constraints of perception. However, this does not preclude that gesture processing relies on a general ability to internally simulate the actions of others (Hostetter and Alibali, 2019) or by the biomechanical constraints of production (Pouw et al., 2020).

That said, evidence that speech and gestures can be integrated both by a fast and a slower mechanism. Obermeier and Gunter (2015) suggests more than one kind of integration. Again, this may be partly determined by how participants approach the listening task. With the design of our stimuli and experiment instructions, we prioritized that the listening task had no explicit focus on the target gestures [further discussed in Nirme et al. (2019)]. There was more than one gesture per video, and no hints as to which was the target word (explicit or implicit). Previous studies have demonstrated effects on reception without explicit attention to gestures. Riseborough (1981) found that participants, who had been informed that they had to do a memory task after watching video sequences of a speaker reciting a list of verbs, were less aware of perceiving gestures accompanying some words, but were still more likely to recall those verbs compared to uninformed participants. Similarly, Gullberg and Kita (2009) found no overall correlation between overt visual attention to gestures and uptake of information expressed by gestures. On the other hand, it is easy to see how overt attention can be elicited by a listening context such as seeing someone show how to place geometric objects, in which no effect of temporal asynchrony was found on recall by Anikin et al. (2015).

Irrespective of the responsible mechanisms, the main finding is that gesture strokes occurring in synchrony or in advance of associated words positively affects recall. Since no recall cues were present during the retrieval phase, the differences must be related to a stronger or more accessible memory encoding of the associated word. The fact that advanced - but not delayed - gesture strokes improve recall adheres to the phonological synchrony rule and suggests that speech-gesture integration is specifically tuned to temporal patterns in natural production and is not simply a case of general multimodal integration.

### 5.3 Methodological discussion

A few observations must be made regarding our chosen methodology. The item-level analysis reveals a possible limitation of the stimulus design of the current study. We shifted target gestures by a fixed time (±500 ms) relative their original timing. The lower recall accuracy for the delayed gestures group seems to be mostly driven by items where the resulting interval between target gesture onset and stressed syllable in the target words is greater than 400 ms. This suggests that it is important to distinguish manipulations based on resulting intervals, from shifts by a constant time relative the original synchrony (the method used for the current study).

The fact that we did not observe greater differences between our two outcome measures for recall (verbatim and gist) can also be explained by methodological design decisions. First, the way we operationalized verbatim recall implies successful gist recall, leading to great overlap between the two measures. Second, since we were interested in the meaning related to one specific gesture-word pair within a longer sequence, the coders’ tolerance for alternative ways to express the same meaning (gist) was narrow. Perhaps more importantly, the task (recall of “unconnected sentences”) may assign a special role to gestures, and the results may not be generalizable to memory encoding within longer passages with more contextual cues (Cohen and Otterbein, 1992).

Despite these limitations, the general methodology used enhances the relevance of our findings. We believe that to better understand the role of gestures for comprehension, it is important to expand on the common paradigms of singleton-gesture stimuli. Presenting gestures using a digitally animated speaker is a way forward since it allows for precise manipulation of individual gestures within natural sequences of speech and gestures. It allows us to present the same speaker delivering identical speech, facial expressions and non-gestural movement while controlling specific gesture features. Also, in real life gestures are typically not performed or observed in isolation. Being able to precisely manipulate single gestures and present them within natural speech and gesture sequences was a priority in the preparation of stimuli used here [see also Nirme et al. (2019)]. While the meta-study by Hostetter (2011) found no systematic differences in the effect sizes obtained by reception studies using scripted vs. spontaneous gestures, we still see advantages of our approach, particularly in studies specifically looking at implicit effects related to gesture-speech processing.

Moreover, the paradigm involving digitally animated speakers can be elaborated in various ways to open other possibilities. As discussed, our experimental design could be improved by a more controlled stimulus set, e.g., by systematically eliciting gestures in motion captured speakers with more or less redundancy in relation to speech or exploring cross-linguistic differences. The method of manipulating specific gestures within natural sequences could also be used to study how semantically mismatched gestures affect processing. More broadly, digitally animated speakers can enable investigation of gesture communication in three-dimensional virtual, immersive and interactive environments (see Heyselaar et al., 2017) with the ability to fully control and measure aspects of gestures in time, space and form. This could enable experimental study of speech-gesture coordination in conversational contexts, with multiple interacting speakers.

## 6 Conclusion

The results of the current study indicate that the natural temporal coordination in co-speech gesture production (gestures before or with related speech) aids recall of verbal information in reception. This in turn supports the view that speech-gesture integration is special, rather than a case of general multimodal integration. The methodology used—the manipulation of specific gestures within naturalistic, motion-captured sequences—arguably strengthens the validity of the results. It allows us to avoid potential confounds introduced by presenting gestures in isolation and is thus a useful addition to the toolbox of gesture researchers.

## Data availability statement

The raw data supporting the conclusions of this article will be made available by the authors, without undue reservation.

## Ethics statement

The studies were part of a larger research environment under a common ethical approval of the Regional Ethical Review Board at Lund University, Sweden (#408/2014). The studies were conducted in accordance with the local legislation and institutional requirements. The participants provided their written informed consent to participate in this study. Written informed consent was obtained from the individual(s) for the publication of any identifiable images or data included in this article.

## Author contributions

JN: Conceptualization, Data curation, Formal analysis, Methodology, Software, Visualization, Writing – original draft, Writing – review & editing. AG: Conceptualization, Funding acquisition, Project administration, Resources, Supervision, Writing – review & editing. MH: Conceptualization, Formal analysis, Methodology, Software, Supervision, Writing – review & editing. MG: Conceptualization, Funding acquisition, Methodology, Project administration, Resources, Supervision, Writing – review & editing.

## Footnotes

^1^In the remainder of this paper, the term’ advanced’ will be used to indicate gestures that have been pre-posed relative to their original position.

^2^https://archive.mpi.nl/tla/elan

^3^https://osf.io/dwuq7/files/osfstorage
