# Supplementary material for: Early or synchronized gestures facilitate speech recall—a study based on motion capture data
Source: Front Psychol. 2024 Mar 25;15:1345906. doi: 10.3389/fpsyg.2024.1345906 (PMC11002957; doi:10.3389/fpsyg.2024.1345906)
Supplement: Supplementary file 1 [file Data_Sheet_1.docx]

Suppl. 1. Transcripts of speech and descriptions of target getstures in video clips

RH = Right hand, LH = Left hand, BH = Both hands

The numbers in parentheses represent the frequencies of the target words per million in Swedish news and magazine texts, found by queries to the Korp research platform (<https://spraakbanken.gu.se/korp/>).

|  |  | **Speaker 1 (female)** |
| --- | --- | --- |
|  |  |  |
| 1 | Swe (1868.7) | Sylvester tar fram en kikare och kollar, och SER Tweety på andra sidan (typ) gatan, tror jag, i ett hotell eller något sånt |
|  | Eng | Sylvester takes out a pair of binoculars and looks, and SEES Tweety on the other side of the street (kind of), I think, in a hotel or something like that |
|  | Target gesture | pointing RH in gaze direction from close to eyes |
|  |  |  |
| 2 | Swe  (36.9) | Så precis när han kommer (typ) till slutet, av de här trappgrejerna, så FASTNAR han med hjulet i (typ) räls, tror jag det är |
|  | Eng | So right when he arrives to the end (kind of), of these stair things, he GETS STUCK with his wheel in some rails (kind of), I think it is |
|  | Target gesture | pantomining pushing bike steering down BH |
|  |  |  |
| 3 | Swe (127.6) | Och så blir han typ omkullknuffad, TAPPAR sina grejer som han bär på ... Han fortsätter cykla. |
|  | Eng | And then he gets (kind of) pushed over, DROPS his things that he is carrying ... He continues bicycling. |
|  | Target gesture | throws BH up like throwing stuff into the air |
|  |  |  |
| 4 | Swe  (2.6) | Ja han försöker räkna, han gör någon såndär kalkylering på papper. Sen så tar han (typ) en lian och ska SVINGA sig över, åker rakt in i väggen |
|  | Eng | Yes he keeps on counting, he makes one of those calculations on paper. Then he grabs (kind of) a vine and is going to SWING across, goes straight into the wall |
|  | Target gesture | svinga RH sweep from right to left |
|  |  |  |
| 5 | Swe  (77.8) | Nä, först hoppar han lite över sånadära saker. Det är typ som parkour nästan, han hoppar över grejer. Sen tar han sin cykel, cyklar sjukt FORT egentligen bara över saker och blir jagad av han som som springer efter hela tiden |
|  | Eng | No, first he jumps a bit over some of those things. It is kind of like parkour, he jumps over stuff. Then he takes his bike, cycles insanely FAST really, just over stuff and gets chased by him who is running after the whole time |
|  | Target gesture | LH opened and moving outward back of palm out to to the left |
|  |  |  |
| 6 | Swe  (20.2) | Eller han hoppar typ över sånadär räcken och sådana saker och sen så kommer han i ganska SMALA gränder, och så kommer det en snubbe och bär på ett paket |
|  | Eng | Or he kind of jumps over those railings and that kind of things and then he comes to pretty NARROW alleyways, and then a dude comes carrying a parcel |
|  | Target gesture | BH with palms spread vertically facing each other held at constant distance moved following a circular path (bulging right) towards speaker |
|  |  |  |
| 7 | Swe (597.1) | Så cykeln ligger där och rullar, och han FORTSÄTTER kubba iväg. Snubben kommer efter, springer efter |
|  | Eng | So the bike lies there rolling, and he KEEPS bolting away. The dude comes after, runs afterRH palm spread vertically moving from up to down and forward |
|  | Target gesture | RH flicked forwards, pointing straight ahead with all fingers extended. |
|  |  |  |
|  |  |  |
|  |  | **Speaker 2 (male)** |
|  |  |  |
| 8 | Swe (471.8) | Och det var även en sån, vad heter det, som man kunde trycka ner. Alltså en spak på sidan som man trycker ner brödet med. Han har alltså lagt i skivorna, trycker ner brödet och så SLÅR man telefonnumret. Jag vet inte vad det var... |
|  | Eng | And there was also one of those, what is it called, that you could press down, that is a lever on the side that one presses down the bread with. He has thus put in the slices, pressed down the bread and then one DIALS the phone number. I don't know what it was |
|  | Target gesture | LH flicked outward |
|  |  |  |
| 9 | Swe  (1.8) | Idén är väl liksom att man kan vrida på den här, och så har du bilder på olika havsdjur. Bläckfisk, delfin och allt vad det var. Som man kan PROJICERA då genom ficklampan |
|  | Eng | The idea is like to turn this, and then you have images of different marine animals. Octopus, dolphin, and everything it was. That you can PROJECT through the flashlight |
|  | Target gesture | RH moved forward palm down while spreading fingers |
|  |  |  |
| 10 | Swe (1.8) | Just det, så kommer de ut på en ... Det kanske händer något annat före, men de kommer ut på en GÅNGBRO som går över liksom en motorled |
|  | Eng | That's right, so they come out onto ... There might happen something before, but they get out onto a FOOTBRIDGE that like crosses a motorway |
|  | Target gesture | RH moved towards right in straight line palm down |
|  |  |  |
| 11 | Swe (216.6) | Så han ramlar ner, och cykeln bara ramlar FRAMFÖR tåget som kommer |
|  | Eng | So he falls down, and the bike just falls IN FRONT of the train that comes |
|  | Target gesture | RH moved down and forward palm down flat |
|  |  |  |
| 12 | Swe (101.9) | Så han måste också göra det där hoppet ner på tågspåret där, och sen SPRINGA efter den där lilla knarkaren. Eller om han har snott knark eller vad det nu är, jag kommer inte riktigt ihåg |
|  | Eng | So he must also do that jump down to the traintracks, and then RUN after that little junkie. Or if he had stolen drugs or whatever it was, I don't quite remember |
|  | Target gesture | RH moved up and forward, palm out, fingers spread |
|  |  |  |
| 13 | Swe (262.5) | Alltså, du går mot Värnhemstorget och sen till höger då, och sen så tar du till vänster. Och så går du bara framåt PÅ regementsgatan |
|  | Eng | So, you walk towards Värnhem square and then to the right, and then you take to the left. And then you just go ahead ON Regement street |
|  | Target gesture | RH sweep inwards from extreme right towards left |
|  |  |  |
| 14 | Swe  (31.0) | och så kanske höger igen och så kommer du att komma till det här kontorslandskapet, eller inte kontorslandskap men där alla sitter och har sina skrivbord. Och ditt är LÄNGST in i hörnet. |
|  | Eng | and then maybe right again and then you arrive to these offices, or not offices but where everyone sits and have their desks. And yours is the FURTHEST into the corner. |
|  | Target gesture | RH flick / point towards left forward |
|  |  |  |
| 15 | Swe (35.0) | De är på en restaurang då och har käkat frukost. Nu får ni ge lite dricks! Alla SLÄNGER in en dollar var. Och så kommer han den snåla jäveln då, mr Pink, och "Nä, jag tror inte på det" |
|  | Eng | They are at a restaurant then and have had breakfast. Now you have to give some tips! Everybody THROWS in one dollar each. And then comes that cheap bastard, mr Pink, and "No, I don't beleive in that" |
|  | Target gesture | RH small flick from right towards left |
|  |  |  |
| 16 | Swe (437.6) | Det sista man ser är att ... Det är bara EN triangel kvar. Och då börjar han hacka sönder det här huset eller den här triangeln liksom. Och boom, så var det slut! |
|  | Eng | The last thing you see is that ... There is only ONE triangle left. And then he starts to hack apart this house or like this triangle. And boom, it was over! |
|  | Target gesture | RH moved upward and opening |
